# No long‐term effect of 7‐day computer‐assisted cryotherapy treatment after total knee arthroplasty: A randomized controlled trial with 1‐year follow‐up

**DOI:** 10.1002/jeo2.70471

**Published:** 2025-11-03

**Authors:** Gideon Teeuw, Astrid de Vries, Reinoud Brouwer

**Affiliations:** ^1^ Department of Orthopaedic Surgery Martini Hospital Groningen the Netherlands

**Keywords:** computer‐assisted cryotherapy, cooling, knee function, pain, PROMs, TKA, total knee arthroplasty

## Abstract

**Purpose:**

Positive effects on pain and opioid use were found in the first post‐operative week, but long‐term effects are unclear. Since rapid recovery studies show that short‐term benefits may have positive long‐term effects, the aim of this study is to investigate if there are any late effects (until 1 year post‐operative) of computer‐assisted cryotherapy (CAC) after total knee arthroplasty (TKA) on knee function, pain and patient satisfaction.

**Methods:**

A single‐centre non‐blinded randomized controlled trial was performed, comparing a cryotherapy group and a regular post‐operative care group after TKA. The cryotherapy group used computer‐assisted cooling, with instructions for cooling several hours per day for the first seven post‐operative days. Oxford knee scores (OKS) (0–48), Knee Injury and Osteoarthritis Outcome Score‐Physical function Short form (KOOS‐PS) scores (0–100) and numeric rating scale (NRS) pain scores (0–10) were scored pre‐operative and 6 and 12 months post‐operative. Anchor questions about self‐perceived changes in daily functioning and pain (7‐point Likert scale) and patient satisfaction (NRS, 0–10) were obtained at both post‐operative time points.

**Results:**

A total of 102 patients were analyzed, 51 patients in both groups. OKS showed no significant difference between the cryotherapy and the regular care groups after 6 and 12 months (median 37.0 vs. 38.0 after 6 months and 40.0 for both groups after 12 months, *p* = 0.634 and *p* = 0.754). Also, no significant differences were found between groups in KOOS‐PS score, NRS pain score, anchor questions and patient satisfaction.

**Conclusions:**

This study showed that use of CAC for 7 days post‐operative after TKA has no significant late effects (until 1 year post‐operative) on functioning, pain and patient satisfaction. This could imply that the clinical benefits of 7 days of cryotherapy are too short to demonstrate longer‐term effects. Limitations such as non‐blinding and lack of objective outcome measures might have influenced the outcomes.

**Level of Evidence:**

Therapeutic study with level of evidence I.

**Abbreviations**
ADL: activities in daily living
CAC: computer‐assisted cryotherapy
IQR: interquartile range
KOOS‐PS: Knee Injury and Osteoarthritis Outcome Score‐Physical function Short form
MED: medical ethics committee
MUA: manipulation under anaesthesia
NRS: numeric rating scale
OA: osteoarthritis
OKS: Oxford Knee score
PROMs: patient‐reported outcome measures
RCT: randomized controlled trial
ROM: range of motion
SD: standard deviation
TKA: total knee arthroplasty
VAS: visual analogue scale

## INTRODUCTION

Total knee arthroplasty (TKA) is a widely accepted treatment option for end‐stage osteoarthritis (OA) of the knee [16]. In the first weeks and months after surgery, pain can be a limiting factor in rehabilitation after TKA [14, 21, 29]. Therefore, analgesics, including opioids, are widely used post‐operatively, not rarely until many weeks after surgery [10]. Negative side effects and risk of dependency and abuse become more relevant in long‐term usage [12]. Consequently, alternatives for pain reduction are needed. As stated by Martin et al., the application of cold can reduce local blood flow due to vasoconstriction and ensuing the local inflammatory reaction, swelling and heat experience [15]. A number of studies showed a positive effect of cryotherapy in the first post‐operative week [3, 25, 31]. Sadoghi et al. showed improvement in pain and flexion early post‐operatively by the use of computer‐assisted cryotherapy (CAC) [25]. Opioid use can be diminished in the first post‐operative days, as shown by Thijs et al. [31]. Also, Brouwers et al. found a positive effect of CAC during the first post‐operative week in terms of pain reduction and diminished opioid consumption in the first phase of rehabilitation [3]. A recent review from Aggarwal et al. [1] found that several studies show positive results regarding cryotherapy after TKA in terms of knee function and pain reduction, even though they concluded the certainty of evidence was low and more randomized controlled trials (RCTs) are required. Notably, most studies assess the effectiveness of cryotherapy in the acute first few days after surgery, with 12 weeks as the longest follow‐up [31]. Rapid recovery studies are describing positive long‐term effects [19, 26], showing the potential of short‐term benefits in the longer term. To our knowledge, no previous studies were performed regarding pain and functional outcome in the long term after the use of cryotherapy; the long‐term effects of cryotherapy are unknown. The primary aim of this study is to investigate whether there is any long‐term difference in recovery (by means of the Oxford Knee score [OKS]) using CAC after TKA, compared to regular post‐operative care. The secondary aim is to assess if there is a difference in functioning, pain or patient satisfaction after the use of CAC post‐operative compared to usual care, and if there is any difference in complications. This study is a follow‐up of the study performed by Brouwers et al. [3] in our centre. The hypothesis is that the positive effects found by Brouwers et al. on pain and opioid use result in positive effects on recovery in the long term.

## MATERIALS AND METHODS

### Study design

This study is a follow‐up on the single‐centre non‐blinded controlled trial where patients were randomized (based on the week they were scheduled for surgery, odd/even) into a cryotherapy and a regular care group, performed by Brouwers et al. between August 2019 and March 2020 in the Martini Hospital, the Netherlands. The study protocol was approved by the local medical ethics committee (MEC 2019‐11), and informed consent was given by all patients before participation. This study was reported according to the CONSORT guidelines in order to address any methodological concerns as much as possible [20, 27].

### Patients and study groups

Adults with end‐stage knee OA scheduled for a TKA were invited to participate in the study. Exclusion criteria were skin (or other) infections, rheumatoid arthritis, vascular disease, or having a strong preference for one of the two treatment options. Two groups were created, and patients were randomly divided into cryotherapy or regular post‐operative care (control group). Randomization was done using the odd/even week principle, with patients undergoing TKA surgery in odd weeks receiving regular care (control group) and patients in even weeks receiving cryotherapy in addition to the usual post‐operative care. Both groups had no restrictions on using opioids or other pain medication, besides patient‐related factors like allergies or interaction with their own medication use. Post‐operative care consisted of bandage compression for the first 24 h, and starting with full weight‐bearing mobilization and active range of motion (ROM) exercises on the day of surgery. Local analgesia was infiltrated for all patients during surgery. The cryotherapy group received added computer‐assisted cryotherapy (CAC) during the first seven post‐operative days. Computer‐assisted cooling was used. During hospitalization, the ZAMAR ZHC‐MG665A was used, and for the home situation, the smaller CAC system ZAMAR ZT Cube was used. This system was delivered to the patient's home the day before surgery. Both models had pre‐set programs. Patients were instructed to use cooling schedules for the first seven post‐operative days. Schedules started on the day of surgery, and started with cooling right after surgery for 6 h at 6°C. The schedule on day one post‐operative was to cool three times for 4 h a day at 8°C. From Day 2 onwards, cooling was scheduled two to three times a day for 2 h at 8°C–10°C. Details of this cryotherapy care can be found in the study from Brouwers et al. [3]. The cooling schedules are based on the schedules used by Thijs et al. [31], since to our knowledge, there is no agreement yet in the literature for the chosen duration and temperature of the cooling.

### Baseline evaluation

Evaluated baseline characteristics were sex (male/female), age (years), body mass index (BMI) (kg/m^2^), American Society of Anesthesiologists (ASA) score (1–4), Kellgren and Lawrence (KL) score (1–4) and a past of TKA for the contralateral knee (yes/no). Also, post‐operative analgesics schedules (normal, no nonsteroidal anti‐inflammatory drugs [NSAIDs] or other) and blood loss were noted. Normal analgesics schedule meant four times 1000 mg a day paracetamol with addition of NSAIDs, and the possibility to use morphine (short‐ and/or long‐acting).

### Outcomes

The primary outcome measure is the OKS. This 12‐item patient‐reported questionnaire leads to a score of 0–48 points, where 0 indicates poor functioning/high pain and 48 maximal function/no pain. As a secondary outcome, the KOOS‐PS score was used as a 7‐item measurement of physical function, derived from the original Knee injury and Osteoarthritis Outcome Score (KOOS), and can be scored from 0–100 point indicating no difficulty (0) to extreme difficulty (100) [5]. Other secondary outcomes are pain in rest and while loading, measured using the 11‐point numeric rating scale (NRS) scores. Further, the patient's self‐rated health score was evaluated by the EuroQol visual analogue scale (EQ5D VAS) score (0–100). These questionnaires were scored pre‐operative, 6 months post‐operative and 12 months post‐operative. In addition, an overall patient satisfaction score from 0 (not at all satisfactory) to 10 (extremely satisfactory) was used to evaluate the outcomes. Anchor questions were scored by a 7‐point Likert scale, scoring improvement or worsening in activities of daily living and pain compared to the preoperative situation. Satisfaction scores and anchor questions were only scored at 6 and 12 months post‐operative. Possible complications like prolonged wound leakages (>4 days post‐operative), wound healing impairment, prosthetic joint infection and thromboembolic events were scored and evaluated until 1 year post‐operative. No extra control moments or questionnaires were done besides the regular follow‐up.

### Statistical analysis

Descriptive statistics were used to present the data. Means and standard deviations (SDs) were presented for interval data that were normally distributed; medians and interquartile ranges (IQRs) were used when data were not normally distributed (for all outcome measures: skewness of the data). Categorical data were presented using frequencies and percentages. Differences in baseline characteristics between groups were analyzed using an independent samples *t* test, a Mann–Whitney *U* test for the non‐normally distributed variables and a chi‐square test for categorical variables. Differences in patient‐reported outcomes between groups were analyzed using a linear regression analysis, adjusted for potential confounding variables (gender, age, BMI, ASA score, KL score and baseline values). SPSS v.25 (IBM SPSS Statistics for Windows, v.25.0: IBM Corporation) was used, and a *p* value below 0.05 was considered statistically significant. Since the original trial was powered on the NRS pain score in rest after 6 weeks, a post hoc sample size calculation was performed. This post hoc sample size was based on the mean and SD of the OKS at 12 months in the usual care group, using the minimal important difference of 5 points for the OKS [24], a power of 90% and an *α* of 0.05.

## RESULTS

### Patient selection

**Figure 1:**
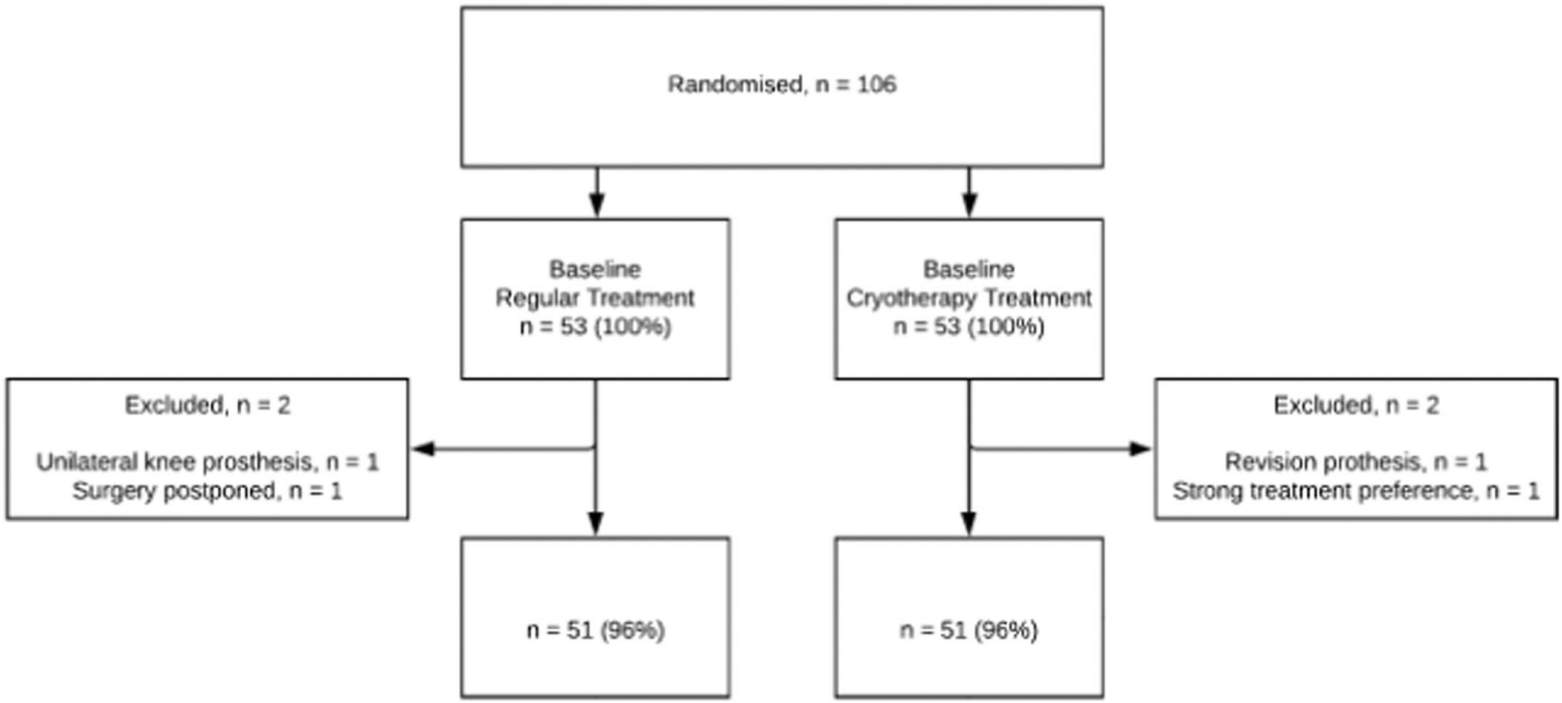
Flowchart of randomization and patient exclusion after randomization.

Initially, 106 patients were included receiving TKA and randomized to cryotherapy or regular care (control group). Four patients were excluded. Two patients received a different type of knee prosthesis (one unicompartmental and one stemmed revision type prosthesis), one patient had a strong preference for the cryotherapy treatment and surgery of one patient was postponed. After exclusion, a total of 102 patients were analyzed, 51 in the cryotherapy group and 51 in the control group. A flowchart of trial participants is shown in Figure 1.

### Baseline characteristics

No differences were found between the two groups in baseline characteristics. Baseline characteristics are shown in Table 1.

**Table 1:** Patient baseline characteristics.

Outcome variable	Reg‐group (*n* = 51)	Cryo‐group (*n* = 51)	*p*
Gender: *n* (%)			1.000
Male	22 (43.1)	22 (43.1)	
Female	29 (56.9)	29 (56.9)	
Age (years): mean (SD)	69.2 (6.8)	69.6 (9.1)	0.815
Body mass index: kg/m^2^ Mean (SD)	31.1 (5.9)	31.5 (5.1)	0.730
ASA score: *n* (%)			0.697
I	3 (5.9)	5 (9.8)	
II	38 (74.5)	38 (74.5)	
III	10 (19.6)	8 (15.7)	
IV	0 (0.0)	0 (0.0)	
Kellgren and Lawrence score: *n* (%)			0.734
0	0 (0.0)	0 (0.0)	
1	0 (0.0)	0 (0.0)	
2	5 (9.8)	3 (5.9)	
3	30 (58.8)	30 (58.8)	
4	16 (31.4)	18 (35.3)	
TKA contralateral knee: *n* (%)			0.208
No	37 (72.5)	31 (60.8)	
Yes	14 (27.5)	20 (39.2)	
Analgesics schedule: *n* (%)			0.786
Normal	30 (58.8)	28 (54.9)	
No NSAIDs	10 (19.6)	9 (17.6)	
Other	11 (21.6)	14 (27.5)	

Abbreviations: ASA, American Society of Anesthesiologists; NSAID, nonsteroidal anti‐inflammatory drug; SD, standard deviation; TKA, total knee arthroplasty.

### Primary outcome

The OKSs showed no significant difference between the cryotherapy and the regular care groups after 6 and 12 months (37.0 vs. 38.0 after 6 months and 40.0 for both groups after 12 months, *p* = 0.648 and *p* = 0.832) (Table 2).

**Table 2:** The Oxford knee score, KOOS‐PS, NRS pain in rest and while loading and EQ5D VAS in both groups at three time points: pre‐operative and 6 and 12 months post‐operative.

	Pre‐operative	6 months FU	1 year FU
	Median (IQR)	Median (IQR)	Median (IQR)
Oxford knee score (0–48)			
Regular	23.0 (12.0)	37.0 (13.0)	40.0 (9.0)
Cryotherapy	25.0 (11.0)	38.0 (9.0)	40.0 (10.3)
*p* a	0.624	0.648	0.832
KOOS‐PS (0–100)			
Regular	44.0 (14.2)	29.7 (16.7)	24.9 (17.0)
Cryotherapy	40.3 (14.2)	29.7 (20.5)	22.0 (25.2)
*p* a	0.654	0.656	0.910
NRS pain in rest (0–10)			
Regular	5.0 (3.0)	0.0 (2.0)	1.0 (1.0)
Cryotherapy	5.0 (4.0)	0.0 (1.0)	1.0 (3.0)
*p* a	0.874	0.619	0.613
NRS pain while loading (0–10)			
Regular	8.0 (2.0)	2.0 (2.0)	1.0 (3.0)
Cryotherapy	8.0 (3.0)	2.0 (3.0)	1.0 (3.0)
*p* a	0.750	0.693	0.924
EQ5D VAS (0–100)			
Regular	70.0 (20.0)	80.0 (20.0)	81.0 (11.0)
Cryotherapy	79.0 (27.0)	80.0 (16.0)	80.0 (15.0)
*p* a	0.127	0.824	0.724

Abbreviations: ASA, American Society of Anesthesiologists; BMI, body mass index; EQ5D VAS, EuroQol visual analogue scale; FU, follow‐up; IQR, interquartile range; KOOS‐PS, Knee Injury and Osteoarthritis Outcome Score‐Physical function Short form; KL, Kellgren and Lawrence; NRS, numeric rating scale.

^a^ Corrected for gender, age, BMI, ASA score, KL grade and baseline value* (*except for comparisons between groups at baseline).

### Secondary outcomes

No significant differences in KOOS‐PS scores were found after 6 and 12 months (*p* = 0.656 and *p* = 0.910). Median NRS pain score in rest was 0.0 in both cryotherapy and regular care groups 6 months post‐operative, also 0.0 for the cryotherapy group 12 months post‐operative and 1.0 for the regular care group 12 months post‐operative. No significant differences were found between the two groups (*p* = 0.619 and *p* = 0.613). Median NRS pain scores while loading showed no significant difference as well (*p* = 0.693 and *p* = 0.924 after 6 and 12 months). The EQ5D VAS scores showed no significant difference between groups (*p* = 0.824 and *p* = 0.724) (Table 2).

**Figure 2:**
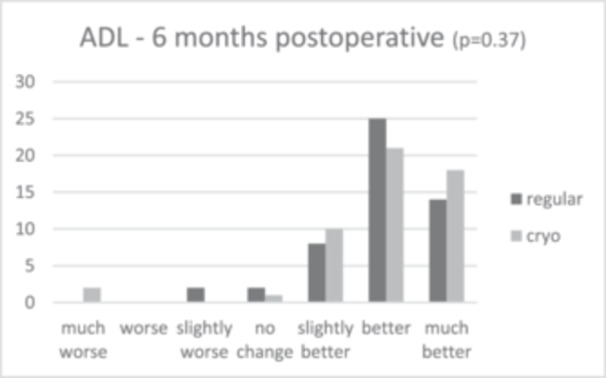
Results of the anchor questions scored by a 7‐point Likert scale, scoring improvement or worsening in activities in daily living (ADL) and pain both at 6 and 12 months post‐operative.

**Figure 3:**
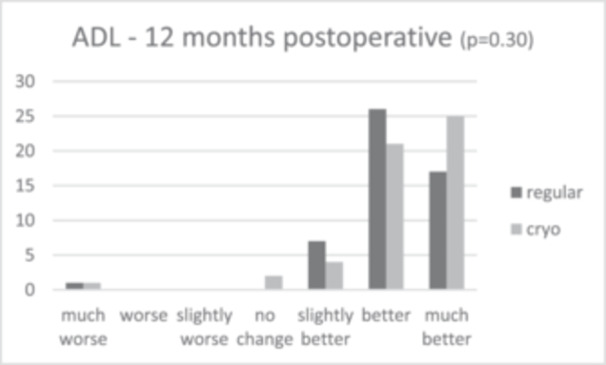
Results of the anchor questions scored by a 7‐point Likert scale, scoring improvement or worsening in activities in daily living and pain both at 6 and 12 months post‐operative.

**Figure 4:**
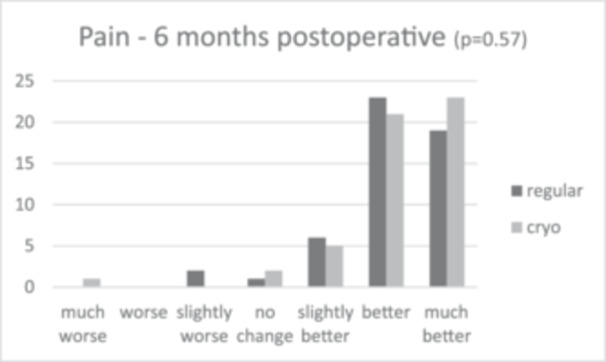
Results of the anchor questions scored by a 7‐point Likert scale, scoring improvement or worsening in activities in daily living and pain both at 6 and 12 months post‐operative.

**Figure 5:**
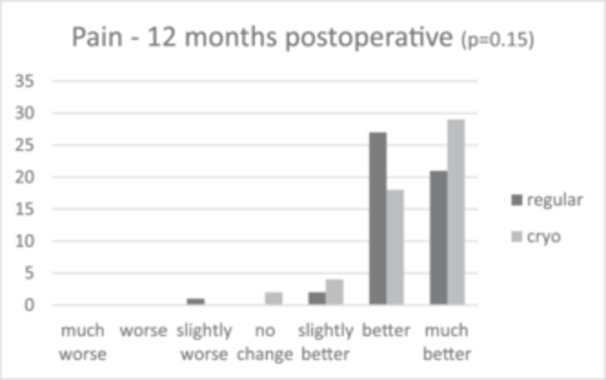
Results of the anchor questions scored by a 7‐point Likert scale, scoring improvement or worsening in activities in daily living and pain both at 6 and 12 months post‐operative.

Patient satisfaction median scores showed slightly greater patient satisfaction in the cryotherapy group (9.0 [IQR = 2.0]) compared to 8.0 (IQR = 2.0) in the regular care group after 6 months, and 9.0 (IQR = 2.0) and 8.0 (IQR = 1.0) after 12 months, respectively. This difference was not significant (*p* = 0.101 and *p* = 0.094). The anchor questions, scoring improvement or worsening in activities in daily living (ADL) and pain 6 and 12 months post‐operative, showed that in all cases, relatively more patients scored a ‘much better’ score for the cryotherapy group for both ADL and pain, as shown in Figures 2, 3, 4, 5. However, no significant differences were found (*p* = 0.37 and *p* = 0.30 for ADL after 6 and 12 months, and *p* = 0.57 and *p* = 0.15 for pain after 6 and 12 months).

Three complications were seen in the cryotherapy group. One patient developed delirium post‐operatively. One patient developed an acute early prosthetic joint infection (with full recovery after debridement, antibiotics and implant retention). One patient had lack of knee function after 2.5 months post‐operative and underwent manipulation under anaesthesia (MUA). One complication was seen in the regular care group, where one patient had prolonged wound leakage. This caused him to stay in the hospital for 2 weeks. No infection occurred in this group.

### Post hoc sample size

The post hoc sample size analysis revealed that with a power of 90% and an *α* of 0.05, based on a mean OKS of 39.2 in the usual care group at 12 months post‐operative and a SD of 7.2, a sample size of 44 patients per group is adequate to determine if there is a difference between groups of 5.0 (minimal important difference) [24].

## DISCUSSION

The primary aim of this RCT was to investigate whether there are any positive long‐term effects on knee function using CAC after TKA. To our knowledge, this is the first RCT evaluating the potential long‐term effects of the use of cryotherapy after TKA. The most important findings of this study are that CAC after TKA for 7 post‐operative days has no significant positive effects on knee function and pain 6 months and 1 year post‐operative. Also, no differences in patient satisfaction and complications were found.

As the application of cold can reduce the local inflammatory reaction and swelling [15], one suggestion why cryotherapy can lead to positive long‐term effects is a ‘head start’ effect in the overall recovery phase. Rapid recovery protocols after knee arthroplasty were already described years ago [2] and have been shown to be effective at reducing post‐operative pain and improving functioning in TKA, also in the long term [19, 26]. Castrodad et al. concluded that early post‐operative rehabilitation after TKA is successful in terms of better ROM and decreased stiffness at 6 weeks post‐operative [6]. This may have subsequent positive effects during the first year of recovery after surgery. Since in our previous study a head start was found, with positive effects on pain in the first post‐operative phase [3], long‐term effects could be expected. No better functional outcomes 6 months and 1 year post‐operative were found, however, in our study, after the use of CAC compared to usual care. These results are in agreement with Thijs et al., who described no differences in knee function 3 months post‐operative in their study. Their study is, to our knowledge, the only study looking at middle‐long term results using CAC after TKA [31]. Wyatt et al. described in their systematic review that cryotherapy appears to have no benefit on ROM and swelling at any point post‐operative [32]. Also, in our previous study, no beneficial effects in knee function were measured with both physical examination tests (ROM and Timed Up and Go) and questionnaires (Work, Osteoarthritis and joint‐Replacement Questionnaire, Knee injury and Osteoarthritis Outcome Score) in the first 6 post‐operative weeks [3]. These results suggest that the use of cryotherapy in the first post‐operative week after TKA has no short‐ or long‐term beneficial effect on functional recovery. A possible explanation why CAC did not show long‐term benefits in our study, might be that our cooling period was too short. In our study, cooling was performed for only 7 days, while knee reactivity seems to be present up to at least 6 months after TKA surgery, according to proven higher temperatures of the knee [9]. Longer post‐operative cooling periods might bring positive effects. Also, numerous influencing factors such as comorbidities, environmental factors or minor complications might have influenced recovery over time and may have interfered with the possible long‐term effects of CAC [4, 18].

Secondary findings in our study were that there is no difference in pain using CAC after TKA 6 months and 1 year post‐operative compared to usual care. Brouwers et al. described that CAC has a positive effect on pain control after TKA in the first post‐operative week, which resulted in less opioid escape medication use [3]. This short‐term effect is probably caused by the combined factors of decreased inflammatory mediators, decreased local oedema, and reduced nerve conduction velocity, which may cause less pain using cryotherapy [13]. Ice has already been proven to help manage acute, haemarthrosis‐related pain [8]. Cryotherapy potentially creates a state of vasoconstriction, which may persist long after cooling ends in the local area of treatment [11]. Recent reviews are describing benefits from cryotherapy after TKA on pain and opioid consumption as well, but only short‐term effects were studied [30, 32]. Thijs et al. are also describing a reduction of opioid use during the first post‐operative days. Similar to our findings, they found, however, no differences in pain 3 months post‐operative between their cold and warm groups [31].

### Strengths and limitations

A strength of our study is that it is the first RCT looking at long‐term results using cryotherapy after TKA. A relatively large cohort of patients is included, with full response of the questionnaires at 6 and 12 months post‐operative, and an adequate sample size to determine a minimal important difference in the OKS between groups [24]. A limitation is that in our study, the cooling took place only during the first seven post‐operative days. This cooling period might have been too short to create a head start effect, translating into positive long‐term results. A longer cooling period post‐operatively might bring more positive effects, also in the long term. Another limitation is that no standardized physical examination tests were performed (e.g., knee circumference or knee ROM) in the long term. Although questionnaires are a strong and reliable scale to measure overall recovery [5, 7], a combination with more objective measurements might give a more complete picture [17]. Also, patients, surgeons and researchers were aware of the type of after‐treatment that participants received; therefore, the study was not blinded in any way. Even though we did not find significant differences between our groups, a placebo effect could be present. This may be reflected by the slightly higher proportion of patients scoring ‘much better’ on both anchor questions despite having similar scores on other outcome measures. A final limitation is that since we used a few exclusion criteria (such as having rheumatoid arthritis), results may not be representative of the full patient population as rheumatoid arthritis has a distinct clinical picture with more post‐operative complications after TKA compared to OA patients [22].

### Future research

Future research should focus on optimizing cryotherapy modalities to investigate the potential beneficial effect on post‐operative knee functioning, pain and opioid use after TKA. Yang et al. [33] concluded in their recent scoping review regarding cryotherapy after TKA that the frequency of cold therapy, the balance in temperature and duration of cooling can still be optimized and needs further research. The effectiveness of a longer period of cooling (e.g. multiple weeks) should be the topic of future studies. Furthermore, Quesnot et al. described in their recent RCT that adding dynamic compression to a (standard) cryotherapy protocol provided further benefits, such as pain reduction and faster improvement in knee function [23]. Sadoghi et al described similar results using their new cryotherapy device [25], and Su et al. found positive effects using a cryopneumatic device over ice with static compression [28]. More research should be done to investigate the added effect of compression on the (long term) effect of cryotherapy after TKA, where a combination of patient‐reported outcomes with more objective functional outcome measures would provide a more complete picture of the actual outcome and lead to strengthened clinical relevance of the findings [17].

## CONCLUSION

The results of this study show that using CAC after TKA for the first 7 post‐operative days shows no difference in knee function or pain at 6 and 12 months post‐operative. Future research optimizing cryotherapy modalities and duration should be done to investigate the potential long‐term effects in recovery after surgery.

## AUTHOR CONTRIBUTIONS

All authors contributed to this study. Gideon Teeuw contributed most to the manuscript and literature search. Reinoud Brouwer contributed to the manuscript and literature search. Astrid de Vries verified the analytical methods and contributed to the manuscript and literature search.

## CONFLICT OF INTEREST STATEMENT

The authors declare no conflicts of interest.

## ETHICS STATEMENT

The study protocol was approved by the local medical ethics committee (MEC 2019‐11). Informed consent was given by all patients before participation.

## Supporting information

CONSORT_checklist.

## Data Availability

The data that support the findings of this study are available from the corresponding author upon reasonable request.
